# Evaluation of a Miniaturized Biologically Vascularized Scaffold *in vitro* and *in vivo*

**DOI:** 10.1038/s41598-018-22688-w

**Published:** 2018-03-16

**Authors:** Sebastian Kress, Johannes Baur, Christoph Otto, Natalie Burkard, Joris Braspenning, Heike Walles, Joachim Nickel, Marco Metzger

**Affiliations:** 1 University Hospital of Würzburg, Chair of Tissue Engineering and Regenerative Medicine, 97070 Würzburg, Germany; 2 University Hospital of Würzburg, Department of General, Visceral, Vascular and Pediatric Surgery, 97080 Würzburg, Germany; 3 Fraunhofer Institute of Silicate Research ISC, Translational Center for Regenerative Therapies, 97070 Würzburg, Germany

## Abstract

In tissue engineering, the generation and functional maintenance of dense voluminous tissues is mainly restricted due to insufficient nutrient supply. Larger three-dimensional constructs, which exceed the nutrient diffusion limit become necrotic and/or apoptotic in long-term culture if not provided with an appropriate vascularization. Here, we established protocols for the generation of a pre-vascularized biological scaffold with intact arterio-venous capillary loops from rat intestine, which is decellularized under preservation of the feeding and draining vascular tree. Vessel integrity was proven by marker expression, media/blood reflow and endothelial LDL uptake. *In vitro* maintenance persisted up to 7 weeks in a bioreactor system allowing a stepwise reconstruction of fully vascularized human tissues and successful *in vivo* implantation for up to 4 weeks, although with time-dependent decrease of cell viability. The vascularization of the construct lead to a 1.5× increase in cellular drug release compared to a conventional static culture *in vitro*. For the first time, we performed proof-of-concept studies demonstrating that 3D tissues can be maintained within a miniaturized vascularized scaffold *in vitro* and successfully implanted after re-anastomosis to the intrinsic blood circulation *in vivo*. We hypothesize that this technology could serve as a powerful platform technology in tissue engineering and regenerative medicine.

## Introduction

In most cases of end-stage organ failure, organ transplantation poses the only way to keep patients alive. However, every year the amount of patients urgently waiting for an organ transplantation is increasing and widely exceeds the number of available organs. Thus, with annually worldwide 119,873 transplanted organs documented in 2014 only less than 10% of the global need was met^1^. In the past, the discrepancy between availability and demand of transplantable organs led to intensified research efforts in the field of tissue engineering and regenerative medicine. This resulted in innovative approaches for the generation of tissue engineered organ replacements^2^.

Nevertheless, the tissues which are currently in clinical use are either flat, hollow or highly artificial in structure^3^. Commonly, they lack an *in vivo*-like vascularization limiting their nutrient supply, three-dimensional thickness and complexity, a phenomenon already observed in 1973 by Folkman and Hochberg^4^. In tissue engineering, bio-printing of vascular structures^5,6^ poses a highly promising attempt in copying the native environment for cellular nutrient supply and waste removal. However, the technology is not yet able to generate fully functional capillary structures and clinically applicable vascular vessel systems.

Other approaches in regenerative medicine try to induce pre-vascularized structures *in vivo* to improve tissue integration and functional restoration^7^. Pre-vascularization is induced either by *in vivo* implantation of a proangiogenic scaffold prior cell transfer^8^ or by endothelial cell vascular self-assembly before implantation^9^, which led to promising results of graft integration into host tissues. However, the first strategy mostly needs two surgical interventions and cells are seeded rather loosely onto a pre-vascularized scaffold. The second attempt takes several weeks until the pre-formed vessel-like system is fully connected to the host’s blood system. Therefore, both approaches limit clinical applicability since larger tissues ideally require an immediate adequate perfusion with nutrients and oxygen.

An attractive alternative is the utilization of xenogeneic decellularized extracellular matrices isolated from small and large animals^10–14^. These can serve as powerful tools for various medical applications without evoking immunogenic host responses^15–17^. With retained vascular structures a framework is provided to be repopulated with autologous endothelial as well as tissue specific cells^10^ that can as such be implanted back into the patient^18–20^. The preservation of feeding and draining vessels as well as the capillary tree ensure a sufficient nutrient supply for *in vitro* maturation as well as an immediate connection to the patient’s blood circulation. Depending on the size of the scaffold even an implantation into small animal models seems possible^12^.

In this work, we have adapted a protocol for the generation of a biologically vascularized scaffold by decellularization of rat intestinal segments and preserved arterial and venous pedicles. We first applied an *in vitro* perfusion culture in a bioreactor system as maturation phase prior to *in vivo* anastomosis and immediate connection to the rat blood circulation supplying for up to 30 days. Evaluation of the scaffold and its harboring cells *in vitro* and *in vivo* proves the concept of its applicability as platform technology for successfully transplantable artificial organ generation.

## Results

### Characterization of a rat miniaturized biological vascularized matrix (mBioVaSc)

**Figure 1:**
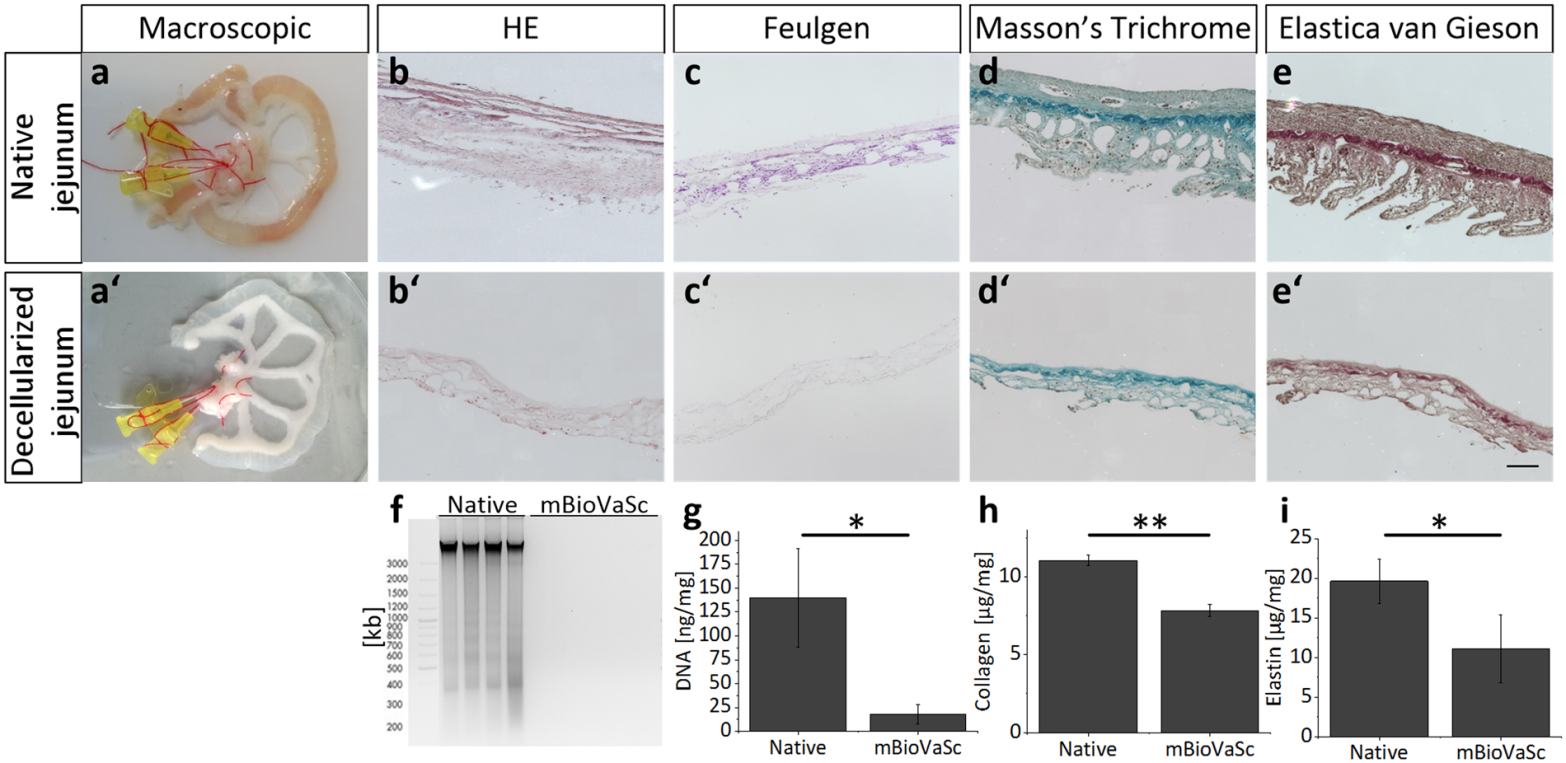
Qualitative and quantitative characterization of the native and acellular rat jejunal segment by histological and biochemical analysis. Segments of rat intestine were cannulated, explanted and subsequently decellularized. (**a**) Macroscopic appearance of the cannulated jejunal segment is shown right after explantation (**a**) and after decellularization (**a’**). (**b**–**e**) Histological analysis of the native rat jejunal segment (**b**–**e**) in comparison to the decellularized mBioVaSc (**b’**–**e’**): HE overview staining (**b**,**b’**); Feulgen reaction denoting DNA residues (**c**,**c’**); Masson’s Trichrome staining revealing residual collagen and ECM components (**d**,**d’**); Elastica van Gieson reaction showing elastic fibers within the matrix (**e**,**e’**). Scale bar, 100 μm. (**f**) Gelectrophoretical separation of DNA fragments before and after decellularization. (**g**) Quantitative determination of the DNA content in the native and decellularized tissue. (**h**,**i**) Quantification of the major ECM components: Collagen (**h**), and Elastin (**i**). Error bars, mean ± s.d. (*p < 0.05, **p < 0.01), (n = 3).

Based on our previous experience with decellularized porcine jejunal segments (the so called BioVaSc-TERM®), homologue segments of the rat intestine were explanted in order to generate a miniaturized biological scaffold with similar characteristics^10,18,19,21^. Accordingly, the native rat vascular system was retained and cannulated via an afferent artery and an efferent vein, respectively (Fig. 1a).

After a multi-step decellularization process (details see methods) the tissue became macroscopically transparent (Fig. 1a’). The structural matrix integrity, however remained widely intact as shown histologically (Fig. 1b–e,b’–e’). We confirmed the rat DNA removal qualitatively via Feulgen staining (Fig. 1c,c’), by fragment size distribution via agarose gel electrophoresis (Fig. 1f), and quantitatively by UV/VIS spectroscopy (Fig. 1g). Genomic DNA extracted from native rat tissue appeared as a strong band with low electrophoretical mobility corresponding to undigested genomic DNA with only small portions fragmented as indicated by discrete, faint bands of sizes which correlate well to single or multiple nucleosomes. In contrast, after the decellularization process no DNA was detected anymore (Fig. 1f) indicating that the DNA levels were reduced below detection limits, confirmed also at increasing exposure times. DNA quantification confirmed the significant reduction of total DNA below 20 ng/mg tissue dry weight after decellularization (Fig. 1g). Besides the removal of cells and cellular remnants verifying complete acellularity, we also analyzed the extracellular matrix integrity and composition. Masson’s trichrome staining depicted in both, the native (Fig. 1d) and the cell free scaffold (Fig. 1d’), a dense matrix structure of cross-linked collagen, although a significant reduction in total collagen I-V content below 30% compared to the native tissue was detected (Fig. 1h). The same accounted for elastin fibers, whose reduction was also significantly reduced (Fig. 1i), however a still dense matrix was observed in histological Elastica van Gieson stainings (Fig. 1e,e’). In conclusion, with our protocol we were able to generate a cell free matrix appearing structurally widely intact.

### **Vascular re-endothelialization and maturation*in vitro***

**Figure 2:**
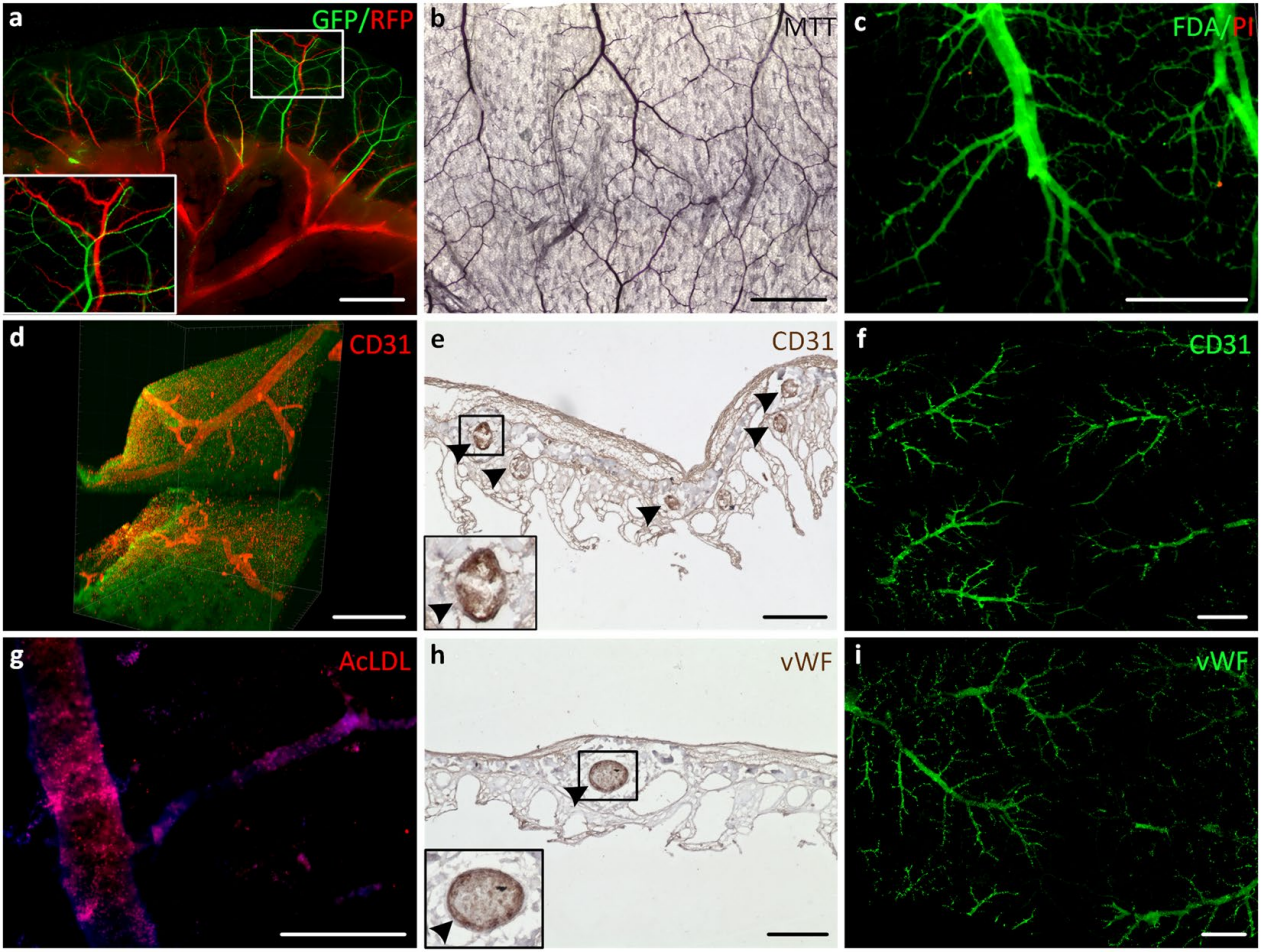
Re-endothelialization of the acellular mBioVaSc’s vascular tree. The preserved vascular system of the acellular mBioVaSc was repopulated with mvECs and cultured in a bioreactor mimicking physiological perfusion. The repopulation of the vessels was determined by live imaging, immunohistochemistry, metabolic activity, viability and functionality displaying branching capillaries in the luminal wall lined with endothelial cells. (**a**) Live imaging of a mBioVaSc segment with ECs expressing GFP and RFP infused through the arterial and venous pedicle, respectively. (**b**) Metabolic activity of EC-seeded mBioVaSc by colorimetric MTT assay. (**c**) Life-dead staining differentiating viable (FDA) and apoptotic/necrotic (PI) endothelial cells. (**d**) Light sheet microscopical visualization of capillaries inside opposing luminal walls. (**e**) Immunohistological detection of the endothelial marker CD31 in a cross section of the luminal revascularized capillary structures (highlighted by arrowheads). (**f**) Whole mount immunofluorescence staining of the EC-marker CD31 on the luminal surface. (**g**) AcLDL uptake by ECs in the capillary bed of the mBioVaSc. (**h**) IHC staining of vWF in a cross section (highlighted by arrowheads). (**i**) Immunofluorescence (IF) whole mount staining of vWF. Scale bars are 3 mm for (**a**); 1 mm for (**b**,**c**,**f**,**i**); 200 µm for (**d**,**e**,**h**); 100 µm for **(g**).

In the next step, we wanted to demonstrate if the maintained structural integrity of the matrix allows repopulation of the vascular network with human cells. To regain a tightly sealed vascular network we therefore re-colonized the residing vascular structures with human endothelial cells (ECs) for 14 days *in vitro*. After initial cell attachment onto the inner walls of the vascular system the vascular tree was perfused with culture medium using a perfusion bioreactor thereby stimulating the cells with *in vivo*-like flow and pressure conditions (i.e. steadily increasing up to 120 mmHg) enabling their functional maturation. Infusing the vascular system with ECs constitutively expressing either GFP or RFP into the arterial and venous pedicle, respectively, demonstrated a dense interconnected capillary network through the luminal matrix discriminating artery and vein lying next to each other as well as artery-venous transitions (Fig. 2a). After 14 days, MTT assay (Fig. 2b) and life-dead staining (Fig. 2c) confirmed viability and vitality of the cells residing inside the vascular system, respectively, which was not significantly altered even after 49 days of *in vitro* culture. 3D light sheet microscopy visualized the vasculature embedded within the luminal matrix (Fig. 2d). Furthermore, we assessed the presence and distribution of typical endothelial markers such as CD31 and von Willebrand factor (vWF) immunohistologically after 14 and 49 days, respectively. Thus, staining on cross sections as well as on whole mount tissues showed a layer of ECs coating the decellularized vessels throughout the vascular system (Fig. 2e,h,f,i). Additionally, we proved the physiological function by demonstrating LDL-uptake of the ECs inside the vascular structures (Fig. 2g).

**Figure 3:**
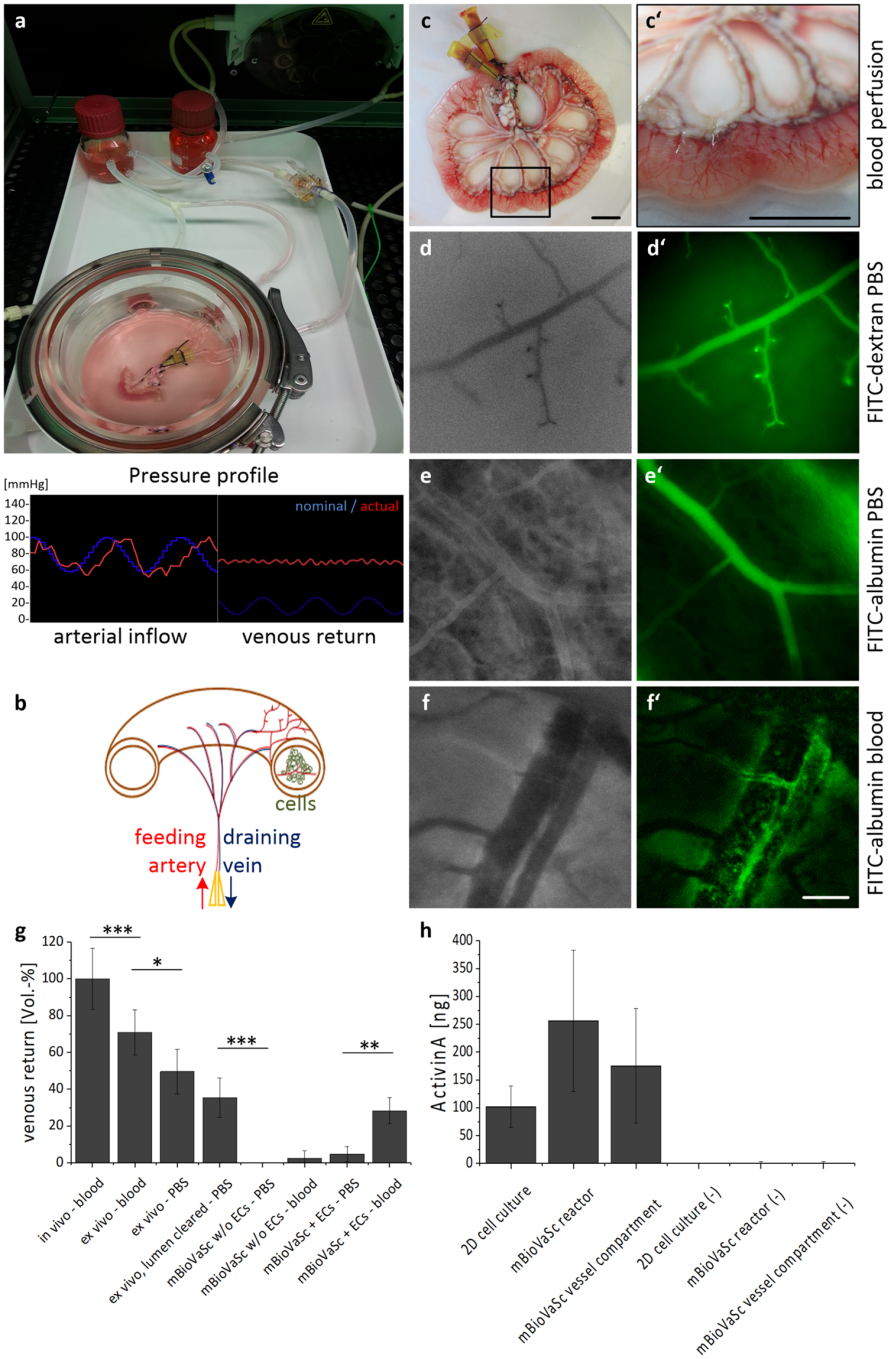
Vascular perfusion of the repopulated mBioVaSc as a platform technology for vascularized tissue culture and drug delivery device. (**a**) Perfusion culture setup for maturation and maintenance culture of the re-endothelialized mBioVaSc in a custom-made bioreactor. Sensing the perfusion-pressure at the arterial inflow and venous return a computer aided system ensured a physiological pressure profile controlling the perfusion pump accordingly. (**b**) Concept of the mBioVaSc showing the preserved cannulated vascular system allowing arterial perfusion and draining through the vein as well as the luminal culture of cells in close proximity to the vascular tree. The pre-vascularization of the vascular system with autologous ECs eases 3D tissue specific cell culture inside the lumen. A bioreactor setup as mentioned above allows for an *in vitro* maturation. The preserved mesenteric vessels enable the microsurgical anastomosis to a patient’s circulation for *in vivo* application. (**c**) The mBioVaSc perfused with heparinized blood *in vitro* depicting macroscopically the tight vascular system. Scale bar, 10 mm. (**d–f**) Phase contrast images of luminal capillaries before fluorescence intravital microscopy with FITC-coupled dextran (**d’**), albumin (**e’**) and albumin in blood (**f’**) perfusion. Scale bar, 100 µm. (**g**) Course of the venous return of the mBioVaSc over the stages from *in vivo* to decellularized to re-endothelialized (n = 3). (**h**) Secretion levels of ActivinA-secreting CHO cells cultured in the lumen of the mBioVaSc and protein distribution through the venous vessel compartment compared to a standard cell culture flask culture. ELISA quantification of supernatant taken from the flask, the bioreactor and the vessel compartment (n = 3). Error bars, mean ± s.d. (*p < 0.05, **p < 0.01, ***p < 0.001).

Culturing the ECs under *in vivo*-like perfusion conditions within the vascular network of the mBioVaSc, the vascular structure could further mature and retrain the vascular tree of the mBioVaSc. To achieve a native pulsatile medium flow the mBioVaSc was connected to a computer-controlled bioreactor system via the vascular cannulated pedicles (Fig. 3a). The pressure on the vascular system was constantly recorded and regulated by automated variance comparison. In contrast to the arterial pulsatile pressure the venous outlet flow was rather constant reflecting physiological conditions of the arterio-venous pressure pattern. Increasing pressure could only be maintained in the vascular system with vessels tightly lined with ECs. Incomplete seeding would result in significant medium leakage and in a rapid pressure loss with no venous backflow. To quantify the medium loss, we measured the perfusion backflow during the whole course of scaffold processing until 14 days of endothelial maturation *in vitro*. Thus, the relative perfusion rate compared to *in vivo* conditions declined from about 70% after explantation to complete leakage after decellularization, but could be partly restored after re-endothelialization to about 30% dependent to the medium condition (PBS or blood) and the inner pressure of the gut lumen e.g. due to feces (Fig. 3g). The reduced backflow suggest that at that time point not all capillaries could be completely sealed by ECs under *in vitro* conditions. Nevertheless, within successfully re-vascularized vessels, intact perfusion could even be seen macroscopically (Fig. 3c). Besides measuring the venous return and macroscopically visualizing the blood-filled vessels from larger feeding/draining vessels (Fig. 3c) towards capillaries in the lumen (Fig. 3c’), we additionally proved the integrity of representative capillaries by real-time intravital imaging of fluorescently-tagged dextran (Fig. 3d’) and albumin (Fig. 3e’) solutions solved either in PBS or blood (Fig. 3f’) until 14 days *in vitro*. All solutions were carefully flushed through the vascular structures and could be repeatedly washed out and re-injected with hardly any leakage detectable even after 15 minutes monitoring. With FITC-albumin solved in blood multiple fluorescent spots could be visualized (Fig. 3f’; Supplementary Video S1) rather than a homogeneous fluorescent solution using the PBS-solved albumin and dextran (Fig. 3d’,e’). Thereby, we could highlight first the influx in an arteriole followed by delayed appearance in the adjacent venule flowing in the opposite direction as in the arteriole. The arterial injected FITC-albumin eventually passed a complete arteriovenous loop and thus approving the continuous integrity of the scaffold’s vascular structures. In conclusion, we demonstrated the significantly recovered functional integrity of the vascular endothelial barrier demonstrated by arterial and venous uni-directional flow through the vessels without significant leakage of fluorescent tracers.

### **Co-culture of tissue specific cells within the mBioVaSc lumen*in vitro***

The concept of the mBioVaSc application is to serve as a platform technology for the generation of implantable and functional organ-specific tissue cultures (Fig. 3b). Well preserved mesenteric gut vessels allow for *ex vivo* perfusion and immediate re-connection to a patient’s vasculature. The capillaries surrounding the former gut lumen facilitate the nutrient supply and waste removal of the tissue culture grown inside the luminal tubular structure. As an *in vitro* proof of concept analysis, we cultured Activin A expressing CHO cells, which were embedded in a collagen hydrogel inside the lumen of the mBioVaSc in close proximity to the re-endothelialized vascular tree (Fig. 3b). Constant viability and vitality was demonstrated for up to seven weeks of culture by live-dead staining and MTT assays (data not shown). We could demonstrate the functionality of the cells and most importantly the ability of the mBioVaSc constructs to function as a drug delivery device by maintaining drug secreting cells in the lumen. Quantification by ELISA revealed that the cells secrete notable more protein inside the lumen as under 2D culture conditions (Fig. 3h). Furthermore, the secreted protein was also detectable in the blood circulation after passing the endothelial cell layer *in vitro*. Interestingly, in the bioreactor vessels the protein concentration reached similar level as measured in the lumen and vessel compartment indicating an equal distribution. However, the permanent perfusion in the bioreactor setup resulted in higher total volume of culture medium indicating that in total more protein was secreted by comparable amounts of cells. For the secreted protein we finally ensured its biological activity in a common cell based assay (data not shown). In conclusion, with this proof of concept study we were able to show long term culture of transgenic cells inside the mBioVaSc.

### **mBioVaSc implantation into rats*in vivo***

**Figure 4:**
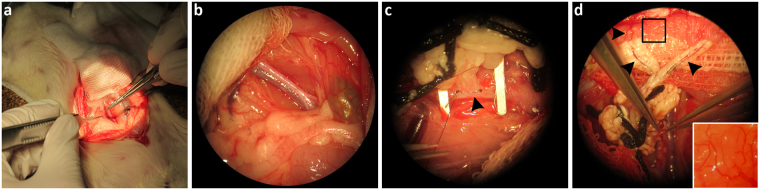
Representation of the mBioVaSc implantation and vascular anastomosis. (**a**) Median laparotomy of the abdominal wall. (**b**) Dissection of the infrarenal part of the aorta abdominalis as well as the caudal vena cava for the subsequent anastomosis. (**c**) Side-to-end anastomosis of the mBioVaSc’s artery and vein with the animal’s abdominal vasculature (highlighted by arrowhead). (**d**) Blood inflow into the mBioVaSc’s vascular system and the capillaries embedded in the lumen (highlighted by arrowheads and inlet) after removal of the clamps congesting the bloodstream during the surgical intervention.

After successfully analyzing the widely intact perfusion and function of the re-endothelialized mBioVaSc over seven weeks in an *in vitro* bioreactor system, the ultimate goal was to test the functionality also *in* *vivo*. As described before, the mBioVaSc was re-endothelialized first and cultured *in vitro* for up to 14 days until sufficient maturation of the vascular system. For implantation, the abdominal wall of anesthetized, immunodeficient adult rats was opened (Fig. 4a) and the caudal part of the abdominal aorta as well as the caudal vena cava (Fig. 4b) were prepared for anastomosis of the preserved mesenteric artery and vein of the mBioVaSc, respectively (Fig. 4c). After anastomosis of both vessels to the rat’s vasculature the surgical clamps (i.e. serrefines) were removed and a blood inflow into the mBioVaSc and its capillaries (Fig. 4d) as well as a venous return was proved. Once opened, it was clearly observable how the blood stream entered the feeding artery flowing through the different branches to the lumen where it spread out into the vast branching capillaries. Excitingly, the partial leakage still observed *in vitro* completely disappeared *in vivo*.

**Figure 5:**
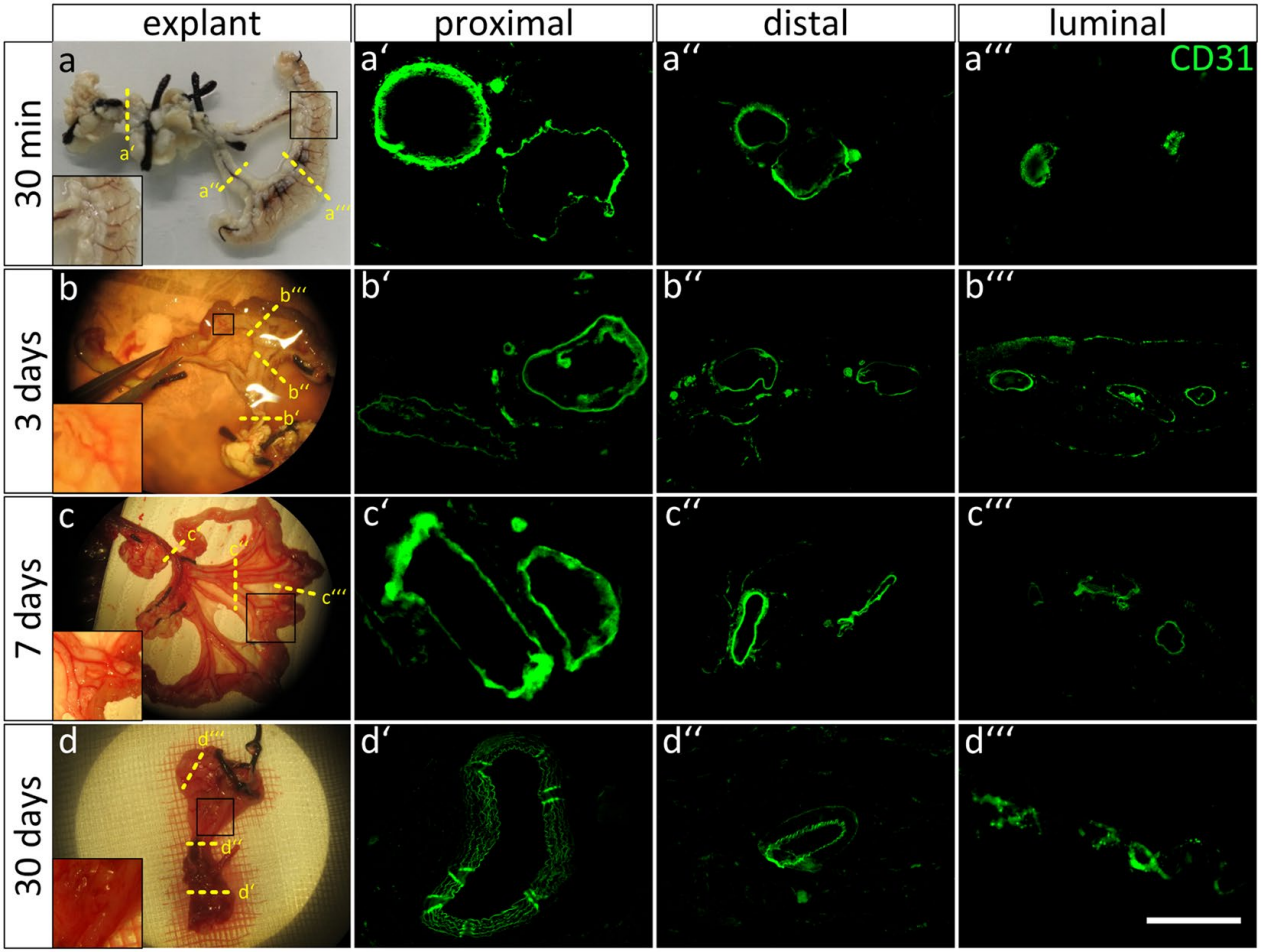
Macroscopic and immunohistochemical analysis of the mBioVaSc after explantation. (**a–d**) Macroscopic representation of the scaffold right after extraction after 30 min (**a**), 3 (**b**), 7(**c**) and 30 days (**d**) *in vivo*. The inlets show a magnification of the vascular tree. The dashed lines indicate the cross sections cut for immunohistochemical stainings for CD31: proximal to the anastomosis (**a’**–**d’**), the distal parts of the feeding vasculature close to the lumen (**a”**–**d”**) and through the lumen (**a”’**–**d”’**). Scale bar, 100 μm.

Over the course of 30 minutes we controlled the perfused scaffold anastomosed to the animal’s circulation (n = 4). In all animals, we detected no visible leakage at any time point and position. We additionally performed manual drainages at different positions of the construct proving the unidirectional blood flow in feeding arterial and draining venous vessels as well as in the capillaries inside the lumen (Supplementary Video S2). In two representative animals, the vascular tightness and the perfusion of the construct through the animal’s circulation could be further confirmed by real-time intravascular fluorescence microscopy after injecting fluorescently labeled albumin into the jugular vein. Thus, the systemically injected tracer could be detected within the vasculature of the anastomosed mBioVaSc within less than 1 minute and was distinctly visible thereafter (data not shown). Immunohistochemical staining confirmed CD31-positive cells present in all the examined vascular compartments of the constructs i.e. in the larger vessel in close proximity to the anastomosis (Fig. 5a’), in the branches leading towards the lumen (Fig. 5a”) and in the luminal capillaries (Fig. 5a”’).

After confirming the tightness of the anastomosis and the vessels intraoperatively, the construct was analyzed upon its vascular integrity in a follow-up study for up to 30 days. After a short recovery period the rats showed normal behavior and weight gain during the following 3 (n = 4), 7 (n = 3) and 30 days (n = 5) until the end of the study. We examined the animals daily throughout the various implantation periods. No altered social, feeding or movement behavior was observed as compared to control animals. In the whole follow-up study the mBioVaSc was tolerated by the rats without any notable complications, incompatibility or rejection except for one animal, which showed weight stagnation and signs of acute abdominal pain after seven days postoperatively and therefore was euthanized. After post mortem explanation it turned out that a progressing ileus had occurred in this animal.

In summary, after 3 days post implantation the implant was easily identifiable embedded in between the rat’s intestine. The macroscopic morphology of the mBioVaSc and its anastomosis was widely unchanged showing no macroscopic signs of necrosis and only minimal fibrotic encapsulation. Also, the blood supply within the scaffold’s vascular tree was clearly detectable from the side of anastomosis to the lumen’s capillary bed (Fig. 5b). The vascular structures were clearly lined with endothelial cells, confirmed by immunohistochemical staining with anti-CD31 antibodies on proximal (Fig. 5b’), distal (Fig. 5b”) and luminal (Fig. 5b”’) cross sections of the explanted mBioVaSc. After 7 days *in vivo*, macroscopically the construct appeared still intact, though surrounded with loose connective tissue adherent to the intestine around the implant (Fig. 5c). Although not as clear as after 3 days, blood filled vessels were visible not only macroscopically but also microscopically by intra-vascular fluorescence microscopy (data not shown). Microscopical studies however only revealed a clearly visible vessel integrity in the larger feeding and draining vessels. The smaller capillaries inside the scaffold’s lumen could not be clearly visualized anymore suggesting a beginning vascular obliteration. The feeding and draining vessels proximal (Fig. 5c’) and distal (Fig. 5c”) to the anastomosis as well as the capillaries within the luminal walls (Fig. 5c”’) were examined immunohistochemically with CD31-positive cells detected in all vessels.

When explanting the mBioVaSc 30 days post implantation it was completely encapsulated by connective tissue and conjoined with the surrounding intestine, which had to be removed first in order to identify the mBioVaSc (Fig. 5d). The anastomosis was still intact but we detected no visible blood flow anymore, even in the larger vessels. The dense connective tissue surrounding the implant also impaired intravascular fluorescence microscopy. Immunohistochemistry staining against CD31 revealed a loosening of the tight endothelial barrier in the larger proximal (Fig. 5d’) and distal (Fig. 5d”) vessels and only a scattered EC detection in the luminal capillaries (Fig. 5d”’).

In conclusion, the microsurgical anastomosis of the scaffold’s vasculature *in vivo* could be successfully conducted with an intact arterio-venous-circulation throughout the mBioVaSc. Macroscopically, the construct was progressively enveloped with a connective tissue resulting in a complete fibrotic encapsulation after 30 days. Microscopically, until 30 days *in vivo*, ECs were still visible, although a tight monolayer could only be proven up to day 7 in our study.

**Figure 6:**
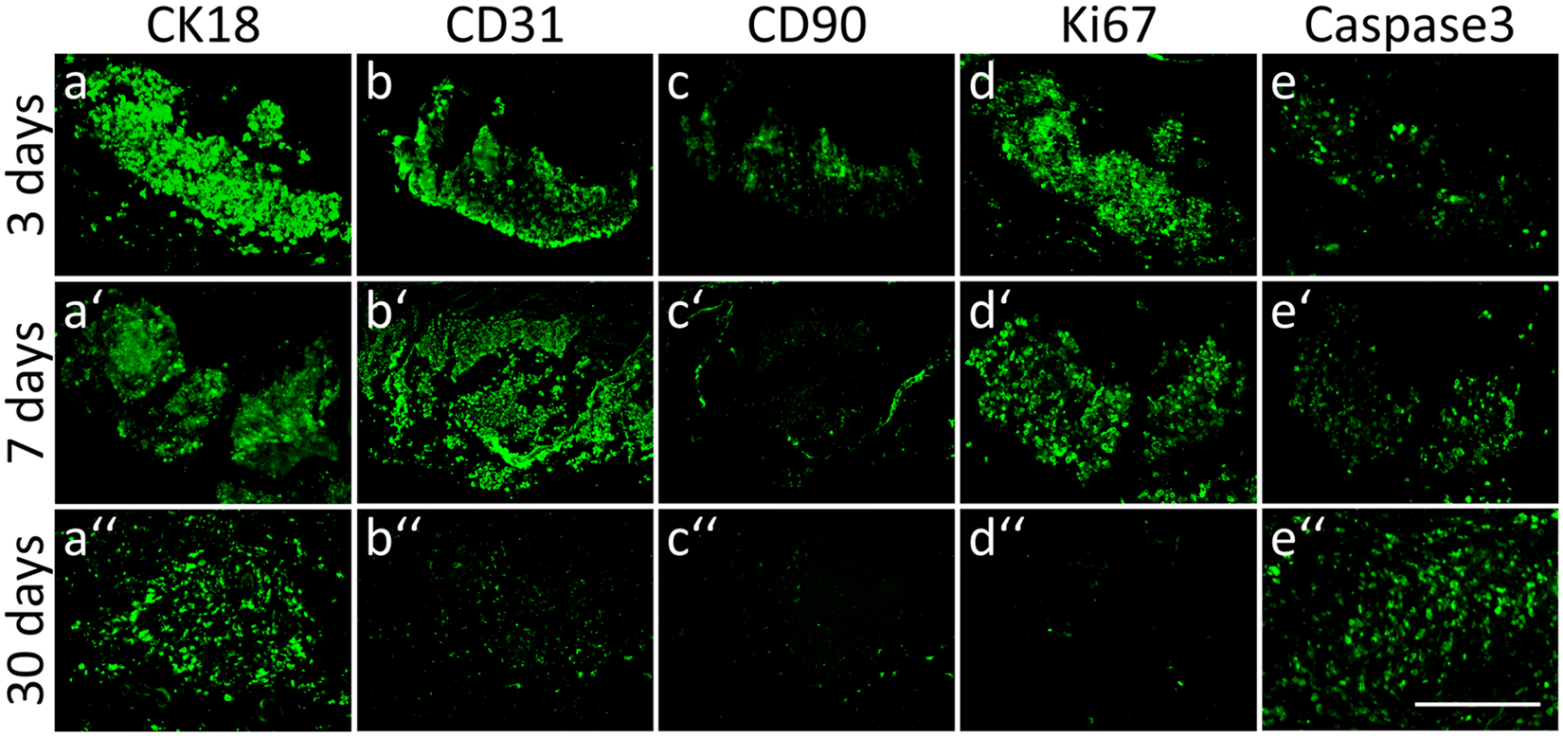
mBioVaSc as platform technology for tissue maintenance *in vivo*. Metabolic highly active liver-like organoids have been generated *in vitro*, transferred into the luminal part of the mBioVaSc and implanted for 3 (**a–e**), 7 (**a’**–**e’**) and 30 (**a”**–**e”**) days. Representation of the organoid-incorporated cell types via immunohistochemical staining for cell type specific markers - CK18 (**a**-**a”**), CD31 (**b**-**b”**) and CD90 (**c**-**c”**) - used for the employed cell types – hepatocytes, endothelial cells and mesenchymal stem cells, respectively. Highlighting proliferative properties of the cells by Ki67 (**d**-**d”**) staining, whereas activated cleaved Caspase3 (**e**-**e”**) was used as a marker for apoptosis. Scale bar, 100 µm.

As proof of concept if a 3D tissue could survive *in vivo*, we finally generated spheroidal liver organoids in an *in vitro* co-culture procedure comprising hepatocytes, endothelial cells, and mesenchymal stem cells as a relevant hepatic tissue graft in regenerative medicine as described before^22^. After mBioVaSc re-endothelialization, the vascular tree should supply the artificial liver organoid cultures, which were introduced into the former gut lumen of the scaffold. The matured tissue construct was subsequently implanted into rats as described above. In a follow-up study, we confirmed the presence of all three organoid cell types immunohistochemically via characteristic markers (CK18 (Fig. 6a-a”), CD31 (Fig. 6b-b”) and CD90 (Fig. 6c-c”)) over a time period for up to 30 days. However, over time a progressive cell death could be observed when comparing the 3 day (Fig. 6a–e) (n = 2), 7 day (Fig. 6a’–e’) (n = 3), and 30 day (Fig. 6a”-e”) (n = 2) cohorts. These observations were confirmed by a decrease in Ki67- (proliferative) (Fig. 6d-d”) and an increase in activated cleaved Caspase3- (apoptotic) (Fig. 6e-e”) positive cells which corresponds to the observation described above i.e. decreasing blood supply over time.

## Discussion

When generating more complex and highly metabolic active organs, functional tissue vascularization is mandatory providing sufficient nutrient and oxygen supply as well as the removal of metabolic waste products due to limited diffusion dependent on the tissue organization and framework. For higher organized, highly metabolic organs with a thick and dense structure the distance of nutrient and oxygen diffusion is even lower, thereby, a dense vasculature is even more important.

A novel interesting approach uses triple cultures of endothelial cells, mesenchymal cells and target cells of interest, which can build pre-vascularized organoid structures *in vitro* and therefore lead to improved *in vivo* survival and function^9^. Furthermore, the use of porcine small intestinal submucosa has demonstrated its viability in a manifold of clinical applications generating an organ *in vitro* serving to guide the cells in growth and spatial organization^10,18–20,23,24^. The intestinal origin allows for an individually adaptable scaffold size without altering its innate properties and advantages. Based on different established decellularization methods for porcine gut a structurally and mechanically stable scaffold with a preserved vasculature can also be prepared from rat intestine^12,13,25^. Pro-angiogenic properties of these scaffolds were already observed *in vivo* in a CAM assay^12,13^. As a consequence culturing ECs in a triple culture setting on a native collagen membrane has been further shown to result in the formation of superficial capillary structures. This artificially pre-formed vascularization promoted functionality of target 3D tissue after anastomosis to the host vasculature *in vivo*^26^. However, the generation of a pre-vascularized scaffold before tissue culture is superior to *in vitro* *de novo* vasculogenesis while tissue establishment. A pre-established functional vascular system can immediately take up the supply function of tissues generated^27^ while a small scale *de novo* vasculogenesis takes 3 to 20 days for maturation until the tissue can be fully supplied^26,28^. A first step towards a functional pre-vascularization of the retained vascular structures of the decellularized scaffold was the partial repopulation with endothelial cells showing evidence of the angiogenic potential *in vitro*^12^.

In this work we have generated a miniaturized biologically vascularized scaffold by decellularization of rat intestinal segments while preserving re-connectable arterial and venous pedicels ensuring anastomosis to a patient’s vascular system (Fig. 4). The rat origin allowed minimizing the necessary cell mass to repopulate the scaffold for the development of smaller implants with shortened maturation and feasible implantation in small animal models compared to established models derived from porcine intestine^10^. The volume of the rat derived tubular scaffold structure to be populated with cells was reduced to about 10% compared to the porcine BioVaSc-TERM®. Finally, the preservation of an extracellular framework and functional vascularization supports cell maintenance and differentiation for more than seven weeks in culture (Fig. 2). Thereby, taking the fenestrated character of the capillaries into consideration, the vascular tightness could be partially restored (Fig. 3g) and allowed perfusion culture of the construct (Fig. 3). Here, we demonstrated the advantage of 3D perfusion culture in terms of drug secretion. While in all reservoirs both in 2D and 3D the concentrations seemed to have reached a similar saturation, the total amount secreted was still higher in the bioreactor setup presumably induced by the perfusion and therefore constant medium exchange. The accumulated total protein in the vascular structures was 75% higher and in the systemic perfusion even 150% higher than in a static 2D culture (Fig. 3h). Moreover, we showed the luminal secreted protein has been transferred into the vasculature and circulated throughout the capillary system, accomplishing an important premise for the use as drug delivery system *in vivo* in that the secreted protein can pass the endothelial barrier and be distributed throughout the blood system.

The lining of the vascular structures with a functional endothelial layer allowed for *in vivo* implantation with an unobstructed blood flow through the implant as a segment of the animal’s circulation (Fig. 4). The proof of concept of the anastomosis and the functional perpetuation of the scaffold inside the rat’s abdomen proved the feasibility and usability of the scaffold as an implantable device fully connected to the patient’s circulation. The tightness of the endothelialized scaffolds vascular tree prevented leakage, acute thrombosis or rejection and supply of organoid-like structures embedded in the scaffold’s lumen. Yet, long term implantation resulted in a fading endothelialization and continuously dying organoids as well as fibrotic encapsulation. Even though an overgrowth of the implant with connective tissue should not hamper its integration as long as the anastomosis stays intact. It could also be that the surrounding tissue potentially supports further vascular ingrowth over time, which needs to be proven in long-term studies. As observed, enveloping the implant by a surgical mesh protected the scaffold from direct encapsulation by connective tissue but on the other hand excluded an ingrowth and sprouting of external vascular structures. A potential strategy to prevent initial acute encapsulation but allowing long-term integration could be a biodegradable membrane allowing retarded degradation and subsequent gradual tissue integration and vascularization. Furthermore, the outer surface of the construct could be seeded with squamous epithelial cells providing a natural surrogate of internal organs^29^. With our setting, however we experienced increasing apoptosis of the embedded organoid structures (Fig. 6), which was in line with the fading endothelial marker detection in vascular structures (Fig. 5) indicating vessel regression and lack of nutrient supply. An acute thrombosis causing immediate under-supply can be excluded as it should have been detected already in the short term studies that proved functionality of intact circulation. A further explanation for the trend of long-term implant failure might be an imperfect endothelialization of the whole construct causing lack of nutrient supply following thrombosis over time. As shown before, long term insufficient vascular circulation *in vivo* could indeed be caused by blocked capillaries and thereby halted blood supply or vessel regression^30,31^ due to missing molecular stimuli maintaining vascular functionality as well as the structural network stability. In contrast, in our study the structural integrity of the vessels could be maintained *in vitro* for at least two weeks while constantly perfused with vascular growth factors indicating the significance of external stimuli for an intact endothelial barrier. This could also be confirmed in a previous study, demonstrating that functional liver tissue could be maintained subcutaneously by HUVECs and dermal fibroblasts secreting angiogenic factors even after 21 days *in vivo*^32^. In this context, Sasaki *et al*. suggested the impact of secreted angiogenic factors on consistent vascularization and graft supply. However, an increasing decline of fibroblasts was already observed within the first weeks of *in vitro* culture leading to a successive degeneration of vessel structures. This is in contrast to our observations *in vitro* but in line with the vascular regression after several weeks *in vivo* as described above. In future studies, implementation of angiogenic^33^ or survival factors or factor-secreting cells might be beneficial. Furthermore, integration of essential vessel-forming cellular elements such as pericytes^34^ and vascular smooth muscle cells^35^ co-cultured with the endothelial cells might improve functionality and longevity of the implant, both *in vitro* and *in vivo*. Besides improved vessel supply, the implementation of more target cells such as the organoids might additionally function as an angiogenic source stimulating vascular growth.

Since the employed animals had no pathological disturbances we do not know whether the grafts would allow for rescue in short term studies. Implementation of more organoids into the luminal scaffold would principally be possible to develop a lumen-filling organ-like structure. This might balance the demand for steady supply of the graft and might implicate quantifiable metabolic activity of the implemented graft tissue. A follow-up study in a liver -deficient animal model might further urge the preservation of the graft tissue. The liver organoids itself have been characterized in detail before^22^ and it is very likely that the amount of implanted organoids need to be increased in order to detect a systemic effect on liver metabolism or human albumin production. So far, in previous studies metabolic and secretory functionality has only been shown *in vitro*. Implantation into a disease model might still show a rescue if the amount of functional cells or organoids will be increased and the vasculature could be stabilized. Besides, as indicated by the perfusion study (Fig. 3g), the native capillaries in the lumen are permeable when the lumen is cleared. Hence, after reconstitution of the endothelial layer of the vascular system only 30% of the arterial influx returned at the venous pedicle while perfusion culture. The luminal filling might pose a counter pressure to the thin walled capillaries retaining more leakage and thus would lead to a further increase of the perfusion rate. Generating a lumen-filling organ-like structure the sole luminal wall vascularization might have to be further expanded by intraluminal vascularization^36^. Nevertheless, the generation of vascularized hepatic tissue after differentiation and maturation of fetal cells^9^ displays a viable strategy to be combined with the mBioVaSc technology. Dense vascularized mature liver tissue could be generated *in vitro* in the lumen with both vascular networks interconnected and subsequently transplanted. Combining the versatility of the platform with studies on angiogenesis and blood vessel formation^12,26^ the full vascularization of the inner lumen interconnected to the pre-existing vascular structures embedded in the luminal wall seems applicable. Thereby voluminous dense tissue structures might be assembled and sufficiently supplied in long term studies. Furthermore, results of studies on the angiogenic potential of arteriovenous loops^37,38^ could be applied here for a fully functional intra-luminal vascularization, also with other tissues.

Interestingly, the vascular degradation has not been observed in this degree in the porcine derived BioVaSc-TERM® after implantation^18^. Its bigger size of the vascular structures and subsequent differences in pressure dispensation might circumvent vascular obliteration. In addition, further cell types such as pericytes and vascular smooth muscle cells could be employed to form fully functional vessels to support the vascular tightness and long term perfusability.

In conclusion, with our study the first basis for complex tissue engineering was prepared by establishing a biologically vascularized scaffold, perfusable for maturation *in vitro* mimicking *in vivo* conditions. The integration of organoids and secretory cells into the pre-vascularized scaffold connected to the bioreactor system provides sufficient nutrient as well as framework supply for further growth, expansions and functional maturation. In addition, the mBioVaSc was successfully implanted into rats proving the concept to function as an implant. The generated mBioVaSc served *in vitro* as well as *in vivo* as a platform technology for cells and organoids secreting biomolecules or metabolizing substances while the vascularization of the scaffold is connected to the circulation to its recipient. The vascularization ensured the sufficient supply of the cells inside the scaffold as well as provided the transport system to further distribute native or metabolized molecules into the circulation.

This work was combining previous achievements in matrix biology, decellularization, vascularization and tissue engineering. We improved the applicability towards clinical relevance by demonstrating the proof of concept for *in vivo* application. With further improvements concerning long term implantation stability of the mBioVaSc as an implantable platform technology for tissue substitutes in combination with the *in vitro* observed potential to function as a drug delivery device the mBioVaSc might be applicable for cell therapy for any chronic and complex metabolic diseases like diabetes, sarcopenia, osteoporosis or hemophilia. The mBioVaSc could offer an intrinsic reservoir of functional cells with the ability to sense the respective protein levels in the blood and act as a sustained reservoir of secreted factors to regain homeostasis of an impaired balance of growth factors and hormones. Finally, with further improvements in terms of long term culture this advancement would make new therapeutic approaches accessible, surpassing and superseding the benefits of classical pharmaceutical treatments.

## Methods

### Animal welfare compliance

All animals received humane care in compliance with the guidelines by the FELASA, WHO and FDA (WHO-TRS978 Annex3 und FDA-OCTGT Preclinical Guidance) after approval from our institutional animal protection board (registration reference number #2532-2-12, Ethics Committee of the District of Unterfranken, Würzburg, Germany).

### Graft harvesting

Lewis rats (age: 8 weeks; body weight about 200 g) were obtained from Charles River, Sulzfeld, Germany and underwent scaffold harvesting under compliance with ethics review formalities. General anesthesia was induced by continuous isoflurane inhalation-anesthesia. The animals were sacrificed by an overdose of the anesthetic. A median laparotomy was used to isolate a 5–10 cm long segment of the jejunum, including its artery and vein pedicle. Following systemic administration of heparin (100 IE/kg) the feeding artery and draining vein were cannulated with a 24-Gauge catheter and flushed with PBS. Venous backflow was controlled macroscopically. A segment of a sacrificed rat’s jejunum was explanted together with its cannulated feeding artery and draining vein. Residual blood inside the supplying vessels was flushed with PBS-Heparin (100 IE/kg) and the lumen of the segment was cleared of all feces. The specimen were stored at 4 °C until further processing.

### Graft implantation

Graft implantation into the regio abdominalis was conducted in female NIH-Foxn1nu rats (age: 8 weeks) obtained from Charles River, Germany. General anesthesia was induced by continuous isoflurane inhalation-anesthesia with intraoperative analgesia (Carprofen, 5 mg/kg s.c.). The abdominal cavity was opened by median laparotomy. The infrarenal aorta abdominalis and the infrahepatic vena cava were dissected from fat and connective tissue. After clamping the vessels proximal and distal an incision of about 3 mm was made for the subsequent anastomosis. The mBioVaSc’s artery was anastomosed side-to-end to the aorta abdominalis and the scaffolds vein to the vena cava. After examination of the patency of the anastomosis and the pervading of the scaffold with blood the abdominal cavity was closed occluding the abdominal musculature and closure of the skin with sutures.

### Matrix preparation and acellularization

To obtain a cell-free matrix the scaffold was decellularized using a modified method described by DeCoppi^13,25^. In brief, both, the intestinal lumen and the vascular tree were continuously perfused for mechanical removal of cell debris during chemical dissociation of the scaffold’s cellular content. For decellularization the scaffold was subsequently perfused with deionized water 4 °C for 24 h, followed by perfusion with 4% sodium deoxycholate (Sigma) at room temperature (RT) for 4 h, and finally with 1 mg/ml DNase-I (Sigma) at 37 °C for 3 h. Every step was followed by a 30 min PBS wash. After treatment the constructs were sterilized by 25 kGy gamma-irradiation and stored in PBS at 4 °C^39^.

### Matrix characterization

Parts of the decellularized scaffold were fixed in formalin, embedded in paraffin, then sectioned at five microns, and transferred to glass slides for further histochemical stainings. Efficiency of the decellularization process was assessed qualitatively by hematoxylin and eosin (HE) and Feulgen staining. The quantity and length of remaining DNA was assessed using a blood and tissue kit (Qiagen) for DNA extraction. The extracted DNA was analyzed by agarose gel electrophoresis and quantified by incorporation of pico-green. Briefly, total DNA was isolated by phenol/chloroform extraction. The amount of remaining DNA was quantified by PicoGreen (Invitrogen) according to the manufacturer’s protocols. Qualitative histological analyses were performed to visualize the structural preservation of the scaffold and to determine the presence, distribution, and relative quantity of constitutive components of the tissue. Briefly, HE, Masson’s Trichrome, Elastica van Gieson staining were used to examine overall tissue structure, maintenance of collagen, glycosaminoglycan and elastin content organization. Quantification of the structural protein content was conducted using a sircol collagen assay and a fastin elastin assay (both Biocolor) according to the manufacturer’s protocols. All results were compared with native tissues as a control.

### Cell culture

All cells were maintained in a controlled humidified incubator at 37 °C, with 5% CO_2_.

Primary isolated microvascular endothelial cells (EC) for reendothelialization of the vascular structures of the mBioVaSc were cultured in Vasculife (Lifeline Cell Technology) media.

Microvascular endothelial cells were isolated from skin biopsies. Briefly, fat, connective tissue and hair was removed from the skin biopsy. Washed and cut stripes were incubated in Dispase (2 U/ml) for 16–18 h at 4 °C. After Dispase digestion the epidermal layer was detached and discarded. The dermal part was washed with Versene (Life technologies) and subsequently exposed to trypsin digestion for 40 min at 37 °C. Primary cells were scraped from the tissue into pre-warmed Vasculife, filtered through a cell restrainer and seeded for cell expansion. 1% Pen/Strep was added to the culture medium.

For live imaging of ECs, the cells were transduced lentivirally to constitutively express GFP (pCDH-CMV-MCS-EF1-copGFP-T2A-Puro; System Biosciences) or RFP (cloned in same vector in exchange for GFP). Briefly, HEK293T cells were transfected with the desired vector and packaging plasmids and envelope, psPAX2 and pMD2.G, in a 2:2:1 molar ratio with X-tremeGENE9 (Roche) according to the supplier’s instructions. Supernatants containing lentiviral particles were used for EC transduction. GFP and RFP positive cells were isolated by Puromycin selection.

For recellularization detached ECs were injected through the arterial and venous cannulas into the vascular tree of the scaffold. Dependent on scaffold size, 0.5–1 ml of 1 × 10^6^ cell/ml EC suspension were injected per cannula with an infusion rate of about 4 ml/min. Cells were allowed to adhere during a 1 h static incubation followed by a subsequent second injection in both vessels as described before. To allow maturation and functional lining of the endothelial layer on the vascular bed the scaffold was connected to a bioreactor in which the vascular system was perfused dynamically and thereby mimicking physiological blood pressure conditions. For standardization and reproducibility the perfusion in the bioreactor was computer controlled. Temperature, CO_2_ concentration and humidity were monitored and adjusted as well as the dynamic perfusion was controlled in order to achieve physiological blood circulating conditions. After prevascularization of the vascular tree the scaffold’s lumen could be repopulated for co-culturing the organ or tissue specific, metabolic or drug secreting cell types.

Fibroblasts and CHO (ATCC® CCL-61™) cells were used for co-culture within the prevascularized scaffold and cultured in high glucose Dulbecco’s Modifies Eagle Medium (DMEM, Life Technologies) supplemented with 10% FCS and DMEM/F-12 supplemented with GlutaMAX™ (ThermoFisher) and 10% FCS, respectively.

Culturing of the liver cells and organoid formation was carried out as previously described^22^. Briefly, hepatocytes, liver sinusoidal endothelial cells (LSEC) and bone marrow derived mesenchymal stem cells (MSC) were cultured in upcyte® Hepatocyte Growth Medium, upcyte® LSEC Medium (both Medicyte GmbH, Heidelberg, Germany) and MSCGM™ Mesenchymal Stem Cell Growth Medium (Lonza), respectively. For liver organoid formation, cells were mixed in liver organoid growth medium (Medicyte), added to Matrigel™-coated plates and further incubated at 37 °C, 5% CO_2_ and 95% humidity for the formation of liver organoids. Using fine spatulas the spheroidal organoids were carefully transferred into the revascularized lumen allowing cellular integration and nutrient supply.

### Cell viability

Live/dead staining with FDA (Fluorescein diacetate)/PI (Propidiumiodide) (Sigma) was used for determining cell viability/cytotoxicity within the scaffolds. Briefly, cell seeded scaffolds were washed with PBS. Subsequently, a PBS assay solution containing 0.5 µg/ml FDA and 0.45 µg/ml PI was added to the samples incubated at room temperature for 10 sec and immediately evaluated using a fluorescence microscope (Keyence BZ-9000).

### Metabolic activity

Metabolic activity of cells within the scaffolds was examined by a colorimetric assay using NAD(P)H-dependent reduction of 3-(4,5-dimethylthiazol-2-yl)-2,5-diphenyltetrazolium bromide (Serva) to formazan (MTT-assay). For analysis, samples were incubated in cell specific medium supplemented with MTT to a final concentration of 1 mg/mL for 90 min at cell culture conditions protected from light. The assay solution was aspirated and the samples washed before analysis.

### Immunocytochemistry

The re-endothelialized scaffold was prepared for immunohistological analysis by fixation in 4% paraformaldehyde at room temperature for 2 h. Subsequently, the samples were embedded in paraffin and cut into 3 μm sections. Deparaffinized and rehydrated sections underwent heat induced unmasking and 3% H2O2 induced blocking of the cell-intrinsic peroxidase before incubated for 1 h with the primary antibody to detect CD31 (35 ng/ml; DAKO Cytomation) or vWF (120 ng/ml; DAKO Cytomation) For peroxidase-based antibody detection the DCS Super Vision 2 HRP Polymer Kit (dcs-diagnostics) was used and cells covered with DAB solution according to the manufacturer’s protocols. Finally, the samples were counterstained with hemalaun and dehydrated before validation by phase contrast microscopy.

### Validation of the re-endothelialized scaffold integrity

Immunohistochemical stainings were performed to validate the scaffold´s functional integrity after re-endothelialization. In brief, after explantation the scaffold was fixed in 4% paraformaldehyde at room temperature for 2 h and subsequently embedded in paraffin. 5 μm sections were deparaffinized by dehydration and antigens retrieved as per primary antibodies manufacturer’s protocol. The primary antibody was incubated over night at 4 °C. The secondary antibody conjugated with fluorochrome was incubated for 1 h at room temperature. Washing steps were conducted subsequently after each step. All fluorescence imaging was performed on a Keyence BZ-9000 fluorescence microscope. The used antibodies were: CD31 (35 ng/ml; DAKO Cytomation), vWF (120 ng/ml; DAKO Cytomation), CK18 (5.4 mg/ml; DAKO Cytomation), Vimentin (300 ng/ml; Abcam), activated cleaved Caspase3 (2 µ/ml; Abcam), Ki67 (1:100; Abcam), CD90 (1:50; Abcam), Alexa Fluor 488 FITC, Alexa Fluor 647 (1:400; Invitrogen).

### Intravital microcopy

For intravital microscopy the graft was placed under a standard inverted microscope (Zeiss) and perfused with carbogen-gassed PBS solution at 37 °C. To analyze vessel integrity and microvascular permeability real-time fluorescence was detected after infusion of either FITC-coupled dextran (40 kDa, Sigma) or albumin (66 kDa, Sigma) solution in PBS or blood. For the *in vitro* cultured graft, the solution was infused directly into the vasculature via the arterial pedicle. For *in vivo* analysis, the animal was kept under anesthesia while the jugular vein was prepared for infusion with the dye. The vascular perfusion was observed under a fluorescence microscope as described previously^40^.

### Whole-mount staining for light sheet fluorescence microscopy (LSFM)

After fixation the sample and the whole scaffold was immunohistochemically co-stained as stated above and an optical clearing was performed. The clearing was a two-step process involving a 2 h incubation in n-hexane followed by three times 30 min incubation in benzyl benzoate/benzyl alcohol (1/3 v/v)^41^.

### ELISA

Protein quantification was performed with a DuoSet ELISA Development kit (R&D Systems) to detect Activin A in a sandwich ELISA. Briefly, a 96-well plate was coated with the antibody to specifically bind the desired protein. The samples were added for 2 h to bind the antibodies followed by another 2 h of incubation with a detection antibody. In between each step repeated aspiration and washing steps were conducted. Streptavidin-coupled horse reddish peroxidase was added before a substrate solution allowed for substrate conversion until the stop solution ended the enzymatic reaction. The optical density was determined at 450 nm immediately thereafter.

### Low-density lipoprotein (LDL) uptake

Endothelial cells are able to incorporate LDL through receptor-mediated endocytosis. In order to monitor the EC’s metabolic function the cells inside the vessel structure were exposed to 10 µg/ml AcLDL (Invitrogen) for 4 h at 37 °C by infusing the solution through the arterial pedicle of the vascularized scaffold. Nuclei were stained by incubation of 2 drops of NucBlue™ Live ReadyProbes™ (Invitrogen) per 1 ml assay solution for 30 min. A standard fluorescence microscope served for visualization.

### Statistical analysis

All results are expressed as mean ± standard deviation (s.d.) Differences between experimental groups were analyzed using the one-tailed independent samples t-test. A value of p < 0.05 was considered as statistically significant.

## Electronic supplementary material

Supplementary Video 1

Supplementary Video 2
